# Seroprevalence Study of Anti-HBs Antibodies in the General Population of Vojvodina, Serbia

**DOI:** 10.3390/medicina60030436

**Published:** 2024-03-06

**Authors:** Smiljana Rajčević, Snežana Medić, Aleksandra Patić, Nataša Dragnić, Mioljub Ristić, Vladimir Vuković, Vladimir Petrović

**Affiliations:** 1 Institute of Public Health of Vojvodina, 21000 Novi Sad, Serbia; snezana.medic@mf.uns.ac.rs (S.M.); aleksandra.patic@mf.uns.ac.rs (A.P.); natasa.dragnic@mf.uns.ac.rs (N.D.); mioljub.ristic@mf.uns.ac.rs (M.R.); vladimir.vukovic@mf.uns.ac.rs (V.V.); vladimir.petrovic@izjzv.org.rs (V.P.); 2 Department of Epidemiology, Faculty of Medicine, University of Novi Sad, 21000 Novi Sad, Serbia; 3 Department of Microbiology with Parasitology and Immunology, Faculty of Medicine, University of Novi Sad, 21000 Novi Sad, Serbia; 4 Department of Social Medicine and Health Statistics with Informatics, Faculty of Medicine, University of Novi Sad, 21000 Novi Sad, Serbia

**Keywords:** hepatitis B, anti-HBs, anti-HBc, seroprevalence, general population, Serbia

## Abstract

*Background and Objectives*: Hepatitis B (HB) is a major global health problem and a potentially life-threatening disease caused by the hepatitis B virus (HBV). Also, it is an important cause of morbidity and mortality worldwide. Thanks to serological surveys, testing hepatitis B surface antibodies (anti-HBs) allows for serological assessments of their prevalence. The presence of anti-HBs, which protects against HBV infection, can be attributed to HB vaccination or natural HBV infection. The aim of our study was to evaluate the prevalence of HB surface antibodies (anti-HBs) as an indicator of collective immunity against HBV in the general population of the Autonomous Province of Vojvodina, Serbia. In addition, to distinguish whether anti-HBs were induced by the vaccine or by infection, the presence of antibodies against the hepatitis B core antigen (anti-HBc) was tested among those who were anti-HBs-positive. *Materials and Methods*: A total of 3467 residual sera samples, collected according to the specifications of the European Sero-Epidemiology Network 2 (ESEN2) study, from April 2015 to March 2016, were screened for the presence of anti-HBs using a chemiluminescence immunoassay. The difference between categorical variables was tested using the chi-square test. *Results*: Overall, 1870 (53.9%, 95% CI: 52.3–55.6) participants tested positive for anti-HBs. The median age of the study participants was 17 years (IQR 9–35). The anti-HB seroprevalence decreased with age, ranging from 80.7% (95% CI: 78.9–82.4) in the 1–19-year-old group to 16.4% (95% CI: 12.0–20.9) in the ≥60 years’ age group. A total of 71 (3.8%, 95% CI: 2.9–4.7) serum samples were also anti-HBc-positive. Higher prevalence, but not statistically significant, was noticed in women (4.1%, 95% CI: 2.8–5.4) compared with men (3.5, 95% CI: 2.4–4.8) (*p* = 0.542). Also, there was a significant difference across the age groups, where those ≥60 years old had a prevalence of 65.9% (95% CI: 51.9–79.9) and the age category of 1–19-year-olds had just 0.2% (95% CI: 0.0–0.4) (*p* < 0.001). *Conclusions*: This study provides a comprehensive assessment of the anti-HBs seroprevalence of the general population in Vojvodina and provides an opportunity to better shape the national preventive strategy related to HBV.

## 1. Introduction

Hepatitis B (HB) is a potentially life-threatening liver infection caused by the hepatitis B virus (HBV), and it represents a major global health problem. According to the estimation of the World Health Organization (WHO), 296 million people were living with chronic HB in 2019 worldwide, with 1.5 million new HBV infections occurring each year and 820,000 deaths, mostly due to chronic sequelae such as cirrhosis and/or liver cancer [1]. Vaccination against HB contributed to a declining prevalence of HBV infection at the population level as well as a declining incidence of new infections, especially in childhood. In addition to vaccination, the regular use of condoms, screening of blood donors, and the implementation of blood safety strategies have also contributed to a reduction in the HBV transmission. A serological survey of specific antibodies against HBV is proposed with the aim to estimate the cumulative prevalence of asymptomatic infection in the community and to better implement the proposed measures [2,3]. In order to globally reduce new HBV infections by 90% and mortality by 65% by 2030, the WHO adopted a “Global Health Sector Strategy on Viral Hepatitis, 2016–2020” [4]. 

Data from many studies in different populations have shown that protective hepatitis B surface antibody (anti-HBs) titers (anti-HBs ≥ 10 mIU/mL) can persist for many years after primary vaccination, providing protection against infection [5,6,7,8]. It is known that protective anti-HBs titers can become undetectable over time, but this does not necessarily reflect a loss of immune memory, since the immunological memory persists for >20 years, and if exposure occurs in immunized individuals, they are able to activate their immunological mechanisms regardless of their anti-HBs level [5,9].

The incidence rate of acute hepatitis B in Vojvodina, the northern province of Serbia, has a decreasing trend. While mandatory notification of HB in Vojvodina has been conducted since 1978 [10,11,12], the true incidence rate of acute HB remains unclear, with the average reported incidence in Vojvodina in the previous period being 1.2/100,000. The incidence rates of chronic hepatitis B are several times higher, ranging from 0.1 to 5.3/100,000 in the previous period. Also, the largest number of patients with reported acute HB are mostly men in the age group of 30–49 years [10]. 

In Serbia, mandatory immunization against HB for newborn infants born to HBsAg-positive mothers and people at high risk of HBV infection, including healthcare workers (HCWs), started in 1988. In addition, universal mandatory immunization against HBV of all unvaccinated children at the age of 12 (catch-up campaign) and for all newborns was adopted and implemented in 2005 and 2006, respectively [13,14]. The vaccination schedule for newborns is to receive the first dose 24 h after birth; the second dose is administered at the age of one month and the third dose at the age of six months. According to the Rulebook and Immunization Program, the vaccination of school children against hepatitis B was completed on 1 July 2018, and in the following period, scheduled vaccination only for missed years up to 18 years of age was conducted [13]. 

Thanks to serological surveys, testing anti-HBs facilitates a serological assessment of their prevalence. The presence of anti-HBs, which protect against HBV infection, can be attributed to HB vaccination or natural HBV infection. The main aim of our study was to evaluate the prevalence of anti-HBs as an indicator of collective immunity against HBV among the general population of Vojvodina. In addition, to distinguish whether anti-HBs were induced by the vaccine or by previous infection, we also aimed to examine the presence of anti-HBc among the anti-HB-positive population.

## 2. Materials and Methods

### 2.1. Study Population and Survey Design

The population of Vojvodina is about two million people (~27% of the population of Serbia, excluding the Autonomous Provinces of Kosovo and Metohija) [11]. This study was conducted at the Center for Disease Control and Prevention and the Center for Virology of the Institute of Public Health of Vojvodina (IPHV), Novi Sad, Serbia. The study protocol followed the specifications and guidelines of the European Sero-Epidemiology Network 2 (ESEN2) project [15]. The method of recruitment of study participants, i.e., their serum samples, as well as the design and method of this study are described in detail elsewhere [16,17]. In brief, 3467 serum samples were collected in the period between April 2015 and March 2016 as part of a larger serosurvey conducted in order to assess the level of immunity against several vaccine-preventable diseases in Vojvodina’s population. Detailed testing procedures regarding the study participants is presented in Supplementary File S1.

The main serum bank consists of residual diagnostic sera samples collected from patients referred for routine laboratory testing (for check-ups and diagnostic purposes) from the health care centers and hospitals of Vojvodina. The age, gender, and geographic stratification of the serum bank was representative of the population of Vojvodina according to the last available census. Accordingly, approximately 100 samples were collected for each year band in the age group of 0–19 years and around 200 samples for each of the age groups ≥20 years (20–24, 25–29, 30–34, 35–39, 40–49, 50–59, and ≥60), with about an equal number of samples by gender. The samples were anonymized and marked only with the study reference number, the date of sampling, and the participant’s age, gender, and place of residence. Written informed consent was obtained from all study participants or their parents/legal guardians for children <15 years old before blood samples were taken. Samples from recipients of blood or blood products during the past 6 months before sampling and immunocompromised persons were excluded from this study. Vaccine coverage data for HB vaccination were obtained from registers of immunization of the Government Health Care Centers of Vojvodina, as a part of a routine surveillance of mandatory immunization. Notification records of all acute hepatitis B cases reported in Vojvodina between 1978 and 2016 were extracted from the surveillance database of IPHV [10]. 

### 2.2. Serological Testing

All collected sera were analyzed at the Centre for Virology, IPHV, Novi Sad. Sera were stored at −20 °C until tested for anti-HBs using the ADVIA Centaur anti-HB commercial test. Anti-HBs-positive samples were further tested for anti-HBc using the ADVIA Centaur HBc Total Assay (Bayer, Tarrytown, NY, USA) on the corresponding system (ADVIA Centaur), according to the manufacturer’s guidelines [18,19]. These ADVIA Centaur tests are sandwich immunoassays which use direct, chemiluminometric technology. The diagnostic specificity is 100% for the ADVIA Centaur Anti-HBs Assay and 99.75% for the ADVIA Centaur HBc Total Assay. The ADVIA Centaur assays have a diagnostic sensitivity of 99.0% for the ADVIA Centaur Anti-HBs Assay and 100% for the ADVIA Centaur HBc Total Assay. The ADVIA Centaur Anti-HBs immunoassay is used to measure the level of antibodies against hepatitis B surface antigens in human serum and plasma. The presence of antibodies against hepatitis B surface antigens is used to determine immune status to HBV or disease progression in HBV-infected individuals. Furthermore, measuring anti-HB levels can determine either the need for vaccination or the success of a vaccination regimen in achieving protective immunity. During the test, HBsAg is coupled to magnetic latex particles in the solid phase. In the Lite Reagent, the HBsAg is labeled with acridinium ester. Non-magnetic latex particles are added from the ancillary well. The sample is incubated simultaneously with Lite Reagent, solid phase, and Ancillary Reagent. Antibody–antigen complexes will form if anti-HBs are present in the sample. A direct relationship exists between the amount of anti-HB activity present in the patient sample and the amount of relative light units (RLUs) detected by the system. Anti-HB serological status was determined using standard protocols with sample cut-off values determined by the manufacturer, where more than 10 IU/mL is considered a positive result (anti-HB-positive) and less than 10 IU/mL is considered a negative result (anti-HB-negative). All 3467 serum samples were first tested for anti-HBs, and then anti-HB-positive serum samples were tested for the presence of anti-HBc in order to distinguish vaccinated from naturally infected persons. Anti-HBc testing could not be performed on 11 participants (0.6%) due to insufficient serum. We were unable to request new samples from these participants due to the anonymization of all samples prior to analysis, preventing all access to personal information. Therefore, statistical analysis was conducted on the 1859 participants who tested positive for anti-HBs and had enough serum for testing. 

### 2.3. Statistical Analyses

Statistical analyses were performed using the statistical package SPSS software tool (version 22.0) MedCalc for Windows, version 12.3.0 (MedCalc Software, Mariakerke, Belgium). The annual crude incidence (per 100,000 people) of acute hepatitis B was calculated using data from the population census of Vojvodina as the denominator [20]. We used the Normal Approximation Method to calculate the 95% binomial confidence interval for the prevalence of seropositivity. The difference between categorical variables was tested using the Chi-square test. The level of statistical significance was *p* < 0.05 across the analyses. 

### 2.4. Ethical Consideration

This study was approved by the Medical Ethics Committee of the IPHV on 14 May 2015 under the number 01–79/7a as a part of a wider serosurvey on vaccine-preventable diseases in Vojvodina. Before enrolment in this study, participants or their parents or legal guardians (if they were <15 years) were informed about the aims and purpose of the study and their informed consent was provided. Access to anonymized data was restricted to employees directly involved in this study.

## 3. Results

### 3.1. Seroprevalence Survey

This study included 3467 subjects aged between 1 and 83 years with almost equal distribution by gender of participants (M/F = 1:1). The median age of the study participants was 17 years (IQR: 9–35). Out of 3467 tested samples, 1870 (53.9%; 95% CI: 52.3–55.6) tested positive for anti-HBs. The percentage of seropositive anti-HBs decreased with age, ranging from 80.7% (95% CI: 78.9–82.4) in the 1–19-year group to 16.4% (95% CI: 12.0–20.9) in the ≥60 years’ age group. We further tested anti-HBc status in those anti-HB-positive participants and demonstrated a prevalence of 3.8% (95% CI: 2.9–4.7) in this group. Higher prevalence was noticed in women (4.1%, 95% CI: 2.8–5.4) compared with men (3.5, 95 CI%: 2.4–4.8), but the difference was not statistically significant (*p* = 0.542). Also, the age group ≥60 years old had a prevalence of 65.9% (95% CI: 51.9–79.9) while the youngest age category (1–19 years old) had just 0.2% (95% CI: 0.0–0.4) (*p* < 0.001), as presented in Table 1. 

**Figure 1:**
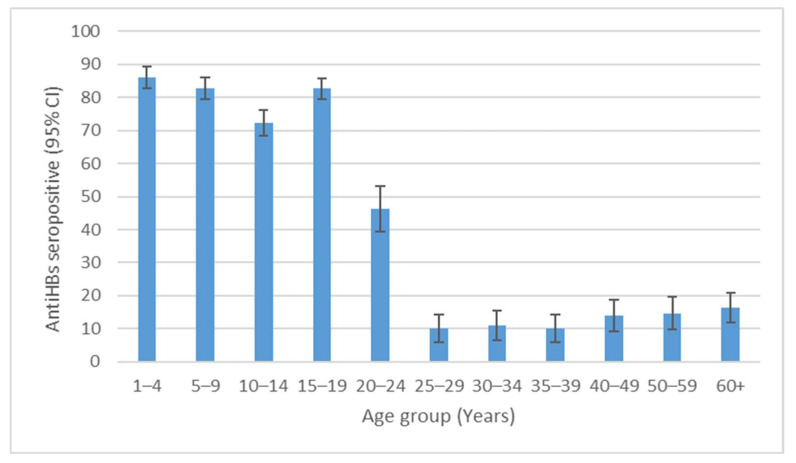
Seroprevalence of anti-HBs by different age groups in Vojvodina, Serbia.

When analyzing age in more detail, we noticed that children aged 1–4 years had the highest (86.0%) anti-HBs seroprevalence. Seropositivity maintained high levels (72.3–82.7%) in pre-school and school-aged children and adolescent age groups (5–19 years), and decreased markedly in the 20–24-year age group (46.3%). The opposite trend, but to a lower extent, was present in the middle and older age groups (>40 years of age) (Figure 1).

**Figure 2:**
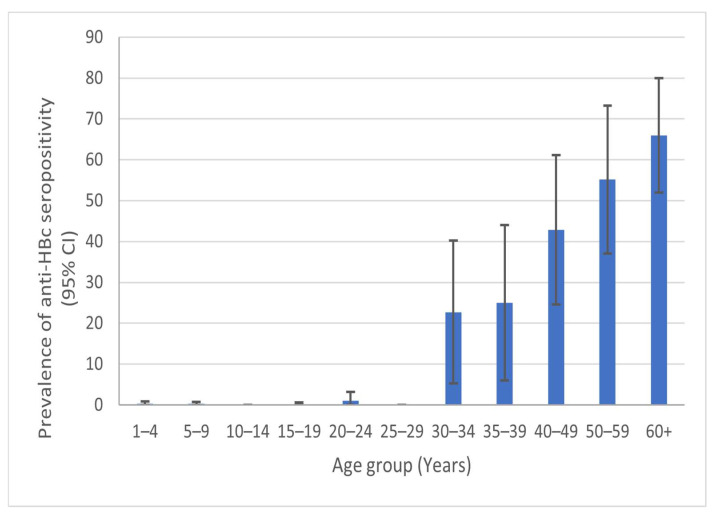
Seroprevalence of anti-HBc in the group of anti-HBs-positive participants by different age groups in Vojvodina, Serbia.

Out of 1859 anti-HBs, a total of 71 samples (3.8%; 95% CI: 2.9–4.7) were anti-HBc-positive. Similarly to the findings regarding the anti-HBs seroprevalence, there was no statistically significant association between the anti-HBs or anti-HBc seropositivity and the gender or area of residence of the study participants. However, anti-HBc seroprevalence increased significantly with age, ranging from 0.2% (95% CI: 0.0–0.4) in the 1–19-year age group to 65.9% (95% CI: 51.9–79.9) in the ≥60 years’ age group of anti-HB-positive participants. Anti-HBc seropositivity remained at a very low level (0.0–1.1%) in anti-HBs-positive children and young adults (<30 years), which then increased sharply in the 30–34 age group (22.7%) and then continued to increase with age to a maximum of 66% in the anti-HBs-positive participants aged ≥60 years (Figure 2). 

### 3.2. Annual Notification of HB Incidence Rates and HB Vaccine Coverage in Vojvodina (1978–2016)

**Figure 3:**
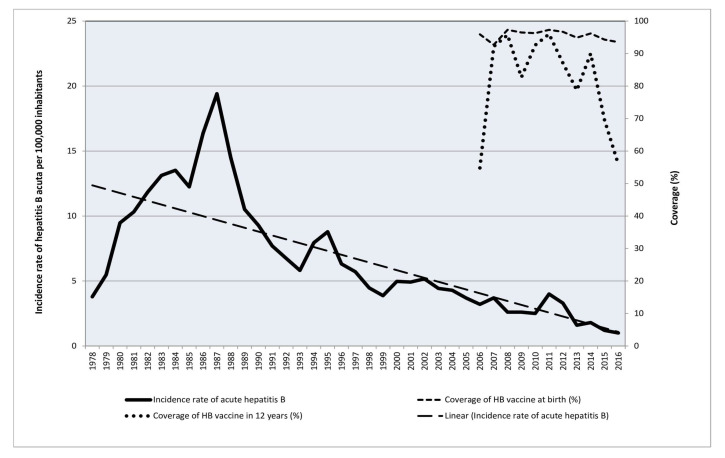
Annual notification incidence rate of acute HB (1978–2016) and HBV vaccine coverage rate (1994–2016) in Vojvodina, Serbia.

The incidence of acute HB in the period of 1978–2016 and HB vaccine coverage rates in the birth cohorts (1994–2016) in Vojvodina are shown in Figure 3.

A declining trend in the acute HB incidence rate in Vojvodina in 1978–2016 was observed, with variation in annual notification rates from 19.4/100,000 in 1987 to 0.9/100,000 in 2016. Based on available immunization data, the coverage rate for HB vaccination in the period of 2006–2016 in infants ranged between 93% and 97%, and in school children aged 12 years, the coverage rate ranged from 55 to 96%. Vaccine coverage in the group of 1–4-year-olds (anti-HB seropositivity of 86.0%) targeted by the anti-HBV vaccination program varied between 94% and 96% in the period from 2014 to 2016. In contrast, the catch-up vaccination campaign for 12-year-olds reported lower coverage (range: 59% to 69%) in the latest observed years (2015–2016, with agglomeration of the population susceptible to HBV. 

## 4. Discussion

To the best of our knowledge, this is the first comprehensive study to assess the anti-HBs in the general population of Vojvodina, Serbia. The results of our study show that 53.9% of participants were anti-HBs-positive (people who acquired immunity from HBV infection or after successful vaccination), and 3.8% of them were anti-HBc-positive, i.e., had natural immunity after HBV infection. The established seroprevalence results cannot be directly compared with the HB vaccination rate due to missing immunization records of the tested subjects. However, the immunity of other anti-HB-seropositive subjects who were anti-HBc-negative was most likely acquired by vaccination. It can be assumed that the obtained anti-HBs seroprevalence is a result of successful vaccination in the cohort of serologically tested subjects who are required to be vaccinated (infants and children vaccinated at the age of 12 years). Vaccination against HB in Serbia has been carried out since 2005/2006, and when it is taken into account that the seroprevalence cross-section was made in 2015–16, this means that the vaccinated generations were covered 18–19 years ago, and this coincides with the high anti-HB levels found in the age group of 1–19 years. This could be a result of vaccine-induced immunity, because otherwise, negative anti-HBc results would not have been found in this specific age group. 

Generally, the seroprevalence of anti-HBs was lowest (up to 20%) during the years before the introduction of the HB vaccine, and it was highest (up to 96%) during the immunization period. Our results also showed that anti-HBc positivity increased with age, clearly indicating an increased risk for exposure to HBV in the pre-vaccine era. Similar results were confirmed by other authors [21,22,23,24]. Moreover, we found that the seroprevalence of anti-HBs in the vaccinated study cohorts (1–22 years of age, i.e., born between 1994 and 2016) was almost 80%, while the prevalence of anti-HBs among unvaccinated cohorts was 18.8%. Seropositivity was significantly higher in one-year-old children (94.5% in 2017) compared to children who received a vaccine against HBV at the age of 12 years (65% in the same year), which might indicate a natural decline in anti-HBs levels over time, as previously demonstrated [25,26]. Data from many studies showed a prevalence of anti-HB seropositivity that was similar to our results and was significantly higher in children, adolescents, and young adults [5,6,27,28]. The highest coverage of HB vaccination and the highest anti-HBs seropositivity was at the age of 1–4 years. One of the explanations is the greater interest of parents in early childhood vaccination compared to vaccination in the teenage period. It is indisputable that the irregular supply of HB vaccine and the reorganization of the health service, which included the abolition of vaccination in schools and the introduction of the institution of an elected pediatrician in Serbia, contributed to the reduced coverage of HB vaccination at 12 years of age. Due to the lack of protective anti-HBs, the risk of HBV infection was higher in the adult age groups (≥20 years). Also, the low prevalence (0.2%) of anti-HBc in 1–19 age group confirms that the anti-HB seropositivity in children and adolescents was most likely the consequence of previous vaccination. There was no statistically significant difference in the anti-HB seropositivity rate based on gender in any age group and region observed in this study, which is similar to other studies [29,30,31,32].

A declining incidence trend of HB is noticeable in other European countries and globally [33,34,35,36,37,38,39,40]. Similarly, a declining incidence rate of acute HB in Vojvodina was observed between 1978 and 2016 in APV. The incidence of acute HB below 1/100,000 recorded in 2016 represents the lowest value since the mandatory notification of acute HB was introduced in APV [10]. A similar trend of acute HB incidence rates was registered in Serbia (1.77/100,000) and, to a three-fold lower extent, in the European Union (≈0.6/100,000) [41,42,43,44]. Compared to acute HB, the incidence of chronic HB in APV was several times higher (3.7/100,000 in 2017) [10], which can be considered as a reflection of the unfavorable epidemiological situation and the lack of universal HB vaccination in the past. Approximately the same rate of chronic HB was also noted at the national level (3.1/100,000 in 2017) [41]. A similar relationship between acute and chronic HB is also registered in some European countries, where the prevalence of chronic HB infection varied between 0.1% and 5.6% [45,46,47,48]. Increased international migration of the population has led to increased incidence of chronic HB in EU/EEA countries [49,50,51]. However, the incidence rate of acute HB remains relatively high only in the high-risk population groups, such as men who have sex with other men (MSM) [52,53,54]. 

Vaccination, along with other control strategies for HBV infection, has contributed to the declining trend of acute HB incidence in the last 20 years (1988–2017) in Serbia (incidence rate decreased from 1.77 in 2017 to 5/100,000 in 2001) [41].

Although universal pediatric immunization against HBV in Serbia was officially introduced in 2002, the implementation started in 2005/2006, and it was at unsatisfactory levels in the first years due to a discontinued procurement of HB vaccines [10,13]. Despite the lack of timely availability of vaccines against HBV in Serbia during 2012–2016, the coverage rate of HB vaccine in newborns reached the desired levels (>90%) [41]. 

The strength of this study lies in the fact that it was population-based with sample collection in accordance with the ESEN-2 methodology [15], as well as in the size of the sample (3467 serum samples correspond to 0.2% of the population of Vojvodina, which corresponds to more than one-quarter of the population of Serbia). Our results, with certain preconditions, could be extrapolated at the national level. Our study also has some limitations. Even though samples were collected in 2015–2016, this remains the only seroprevalence study assessing collective population immunity to HBV conducted in our country since then. Second, not all sera from the study participants were tested for anti-HBc; hence, some of the occult HBV infection cases might be missed. Third, the HB vaccination history of the participants as well as their belonging to a group at an increased risk for HBV (i.e., MSM population, sexual partners of HBsAg-positive patients, hemodialysis patients, intravenous drug users, HCWs, etc.) were unavailable. Therefore, further serosurveys based on a larger national serum bank representative in relation to the age, gender, and geographical distribution of respondents and particularly involving vulnerable population(s) for HBV are warranted. Fifth, there is a possibility that a small portion of anti-HB positivity in the adult participants is a result of vaccination due to their risk of HBV infection (i.e., occupational requirements, partners of an HBsAg-positive person, members of other risk groups, etc.). However, due to the negligibly low coverage of HB vaccine in high-risk groups in the past [10,52], we presume that this limitation did not compromise the main findings of our study. Finally, the ESEN-2-based age stratification study protocol that we followed may have been suboptimal for a detailed assessment of anti-HB seroprevalence in older age groups. Particularly, some of the age categories (i.e., ≥60 years old) had a limited number of participants and thus might not be fully representative of this age group.

## 5. Conclusions

In conclusion, the estimation of the seroprevalence of anti-HBs in Vojvodina provides crucial information for the effective implementation of HB control programs, primarily the promotion/recommendation of HB vaccination in the susceptible age groups identified in this study. The results of our study indicate that there is a high prevalence of anti-HBs seropositivity among children, which can be largely attributed to the implementation of a successful vaccination program in APV. The overall prevalence of anti-HBc increased significantly with age. In addition, appropriate health education programs, such as safe sex and safe injecting procedures for adolescents and the adult population, need to be further developed/adjusted, implemented, and maintained. Further efforts should emphasize the comprehensive inclusion of high-risk groups in continuing education about the benefits of HB vaccination. Considering the facts that protective anti-HB titers can become undetectable over time and that the immunological memory persists for >20 years, we believe that as many as 53.9% of anti-HB-seropositive persons had immunity against HBV infection in our study. In order to better characterize the seroprevalence of HBV and determine the burden of HBV infection in Serbia, additional larger national serosurveys are warranted.

## Figures and Tables

**Table 1:** General characteristics of study population and the level of anti-HBs and anti-HBc seropositivity (of the anti-HB-positive samples).

	Anti-HBs Seropositivity (n = 3467)			Anti-HBc Seropositivity of the Anti-HB-Positive Serum Samples (n = 1859) *		
	n	% Prevalence (95% CI)	*p*-Value ^a^	n	% Prevalence (95% CI)	*p*-Value ^a^
Gender						
Male	1733	53.8 (51.5–56.2)	0.906	930	3.5 (2.4–4.7)	0.542
Female	1734	54.0 (51.7–56.4)		929	4.1 (2.8–5.4)	
Region						
North	924	53.5 (50.2–56.7)	0.364	490	3.5 (1.8–5.1)	0.877
Central	1464	52.9 (50.4–55.5)		768	4.0 (2.6–5.4)	
Southern	1079	55.7 (52.7–58.6)		601	3.8 (2.3–5.4)	
Age (years)						
1–19	2001	80.7 (78.9–82.4)	<0.001	1603	0.2 (0.0–0.4)	<0.001
20–59	1198	17.7 (15.5–19.9)		212	18.4 (13.2–23.6)	
≥60	268	16.4 (12.0–20.9)		44	65.9 (51.9–79.9)	
Total	3467	53.9 (52.3–55.6)		1859	3.8 (2.9–4.7)	

^a^ Chi-square test; * for 11 (0.6%) anti-HBs-positive participants, there was not enough serum to conduct anti-HBc testing, and thus, statistical analysis was conducted on 1859 samples.

## Data Availability

The data that support the findings of this study are available from the corresponding author upon reasonable request.

## Supplementary Materials

The following supporting information can be downloaded at https://www.mdpi.com/article/10.3390/medicina60030436/s1: File S1. Description of the used study protocol (collection, coding, and storing of the samples). Figure S1: Algorithm for hepatitis B serology testing: anti-HBs and anti-HBc [11,15,16,17,55,56].
