# Supplementary material for: Seroprevalence Study of Anti-HBs Antibodies in the General Population of Vojvodina, Serbia
Source: Medicina (Kaunas). 2024 Mar 6;60(3):436. doi: 10.3390/medicina60030436 (PMC10972133; doi:10.3390/medicina60030436)
Supplement: Supplementary file 1 [file medicina-60-00436-s001.zip › medicina-2880235-supplementary.pdf]

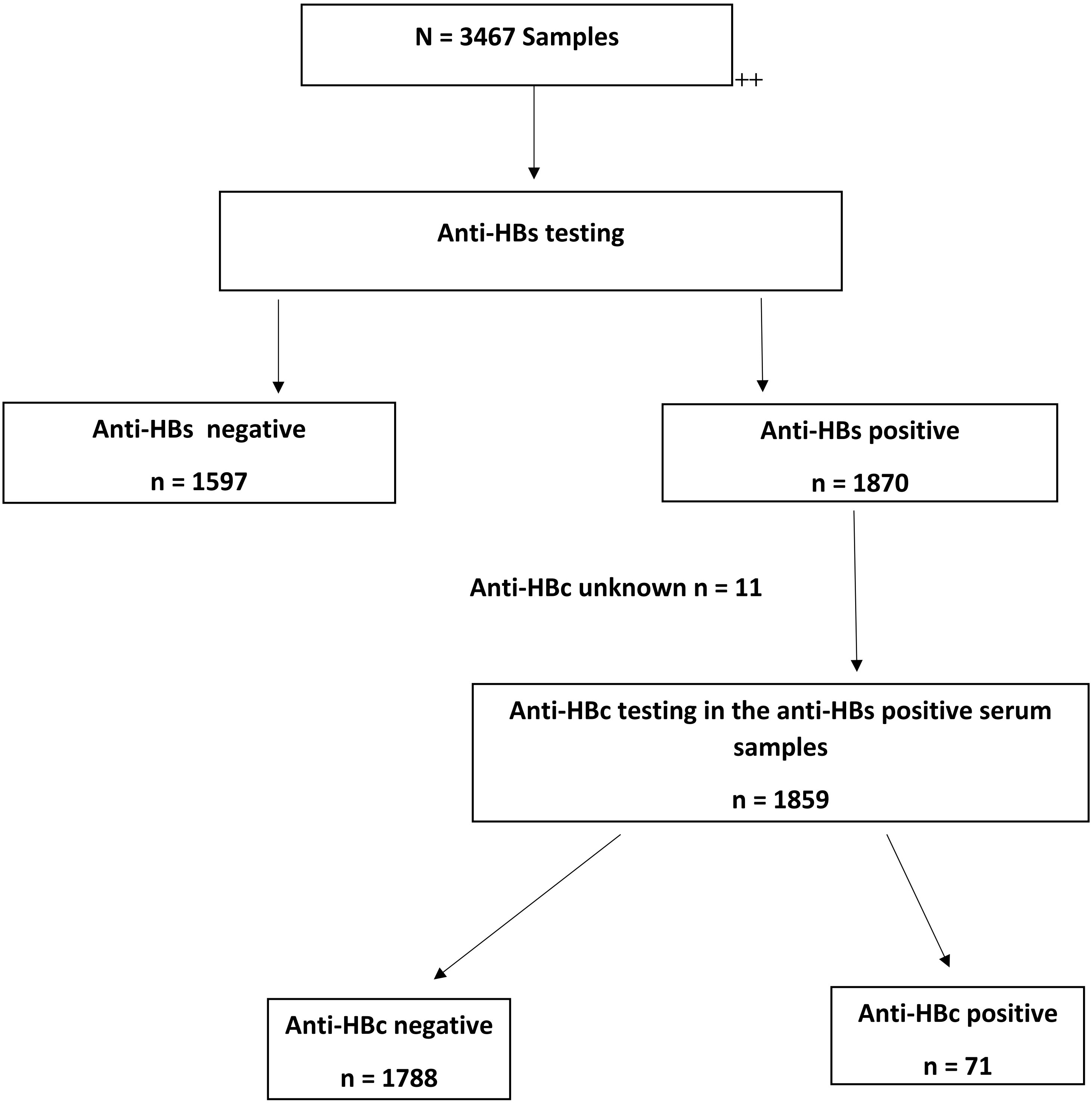


Figure S1: Algorithm for hepatitis B serology testing: anti-HBs and anti-HBc

**File S1.** Description of the used study protocol (collection, coding and storing of the samples).

Serosurvey was conducted between April 2015 and March 2016 in order to assess the level of immunity of the local population against vaccine-preventable diseases. This is the first population-based study that allows for a comprehensive assessment of the current level antibody on HBV in Vojvodina based on the estimation of age-specific HBV seroprevalence. Vojvodina is the northern province of Serbia. The population of Vojvodina was 1,931,809 in 2011, accounting for 26.9% of the population of Serbia, excluding Kosovo. Its borders are with Hungary to the north, Romania to the east, Croatia to the west, and Bosnia and Herzegoovina to the southwest [1]. The population aged under 15 and over 65 in the total population ranged as follows: the percentage of young people (0-14) are 14.3%, while the percentage of the population aged 65 and over are to 21.3%. The average age of the population in the Republic of Serbia is 43.5. thus ranking Serbia among countries with deep demographic age both in Europe and the rest of the world. The main feature of the age-gender structure of the population in the Republic of Serbia is that men prevail in the youth, i.e. women in the middle-aged and aged population [2].

A total of 3467 residual serum samples from patients of all ages were included in the study. Samples were collected according to the specifications of the European Sero-Epidemiology Network 2 (ESEN2) study [3]. The age stratification of the sera was according to the specifications of ESEN2. Sampling method has been described previously by Medić, et al. [4-6]. Available information for each patient included the following: sex, age, area of residence in Vojvodina, and sample collection date. Written informed consent of study participants, or their parents or legal guardians if they were <15 years of age, was obtained. The study was approved by the Medical Ethics Committee of the Institute of Public Health (IPH) of Vojvodina, in accordance to the Declaration of Helsinki of 1975, as revised in 2008.

All the collected sera were stored at −20◦C until tested for hepatitis B. All the sera were tested for anti-HBs, testing with anti-HBs Commercial tests of the ADVIA Centur anti-HBs2, only anti-HBs positiv sera were tested with ADVIA Centaur HBc Total Assay (Bayer, Tarrytown, NY, USA) on the corrensponding system (ADVIA Centaur) according to the manufacturer's guidelines, at the Virology laboratory of IPH of Vojvodina. Obtained results were standardized into ESEN2 units according to the ESEN2 methodology.

1. Statistical Office of the Republic of Serbia. 2011 Census of Population, Households and Dwellings in the Republic of Serbia. [In Serbian/English]. http://pod2.stat.gov.rs/ObjavljenePublikacije/Popis2011/Nacionalna%20pripadnost-Ethnicity.pdf. Accessed 05 December 2022.

2. Statistical Office of the Republic of Serbia. Statistical year book of Republic of Serbia, 2022. [In Serbian/English]. Available from: Accessed 05 December 2022

https://publikacije.stat.gov.rs/G2022/Pdf/G20222055.pdf

3. Nardone A, de Ory F, Carton M, Cohen D, van Damme P, Davidkin I, et al. The comparative sero-epidemiology of varicella zoster virus in eleven countries in the European region. Vaccine. 2007;25(45):7866-7872. doi:10.1016/j.vaccine.2007.07.036

https://www.sciencedirect.com/science/article/pii/S0264410X07008134?via%3Dihub

4. Medić S, Petrović V, Milosević V, Lozanov-Crvenković Z, Brkić S, Andrews N, de Ory F, Anastassopoulou C. Seroepidemiology of varicella zoster virus infection in Vojvodina, Serbia. Epidemiol Infect. 2018 Sep;146(12):1593-1601. doi: 10.1017/S0950268818001619. Epub 2018 Jun 18

https://www.ncbi.nlm.nih.gov/pmc/articles/PMC9507937/pdf/S0950268818001619a.pdf

5. Kafatos G, Andrews N, Nardone A; ESEN2 project. Model selection methodology for inter-laboratory standardisation of antibody titres. Vaccine. 2005 Oct 10;23(42):5022-7. doi: 10.1016/j.vaccine.2005.05.030. PMID: 16002191.

https://pubmed.ncbi.nlm.nih.gov/16002191/

6. Medić S, Katsilieris M, Lozanov-Crvenković Z, Siettos CI, Petrović V, Milošević V, Brkić S, Andrews N, Ubavić M, Anastassopoulou C. Varicella zoster virus transmission dynamics in Vojvodina, Serbia. PLoS One. 2018 Mar 5;13(3):e0193838. doi: 10.1371/journal.pone.0193838. PMID: 29505590; PMCID: PMC5837184.

https://www.ncbi.nlm.nih.gov/pmc/articles/PMC5837184/
